# Comparative analysis of left ventricle function and deformation imaging in short and long axis plane in cardiac magnetic resonance imaging

**DOI:** 10.3389/fcvm.2024.1388171

**Published:** 2024-05-02

**Authors:** Oscar Werner, Duarte Martins, Federico Bertini, Elena Bennati, Dario Collia, Iacopo Olivotto, Gaia Spaziani, Alban-Elouen Baruteau, Gianni Pedrizzetti, Francesca Raimondi

**Affiliations:** ^1^ Pediatric Cardiology Unit, University Hospital Meyer, Florence, Italy; ^2^ Department of Pediatric Cardiology and Pediatric Cardiac Surgery, FHU PRECICARE, Nantes Université, CHU Nantes, Nantes, France; ^3^ Pediatric and Adult Congenital Cardiology Unit, ASST Papa Giovanni XXIII, Bergamo, Italy; ^4^ Pediatric Radiology Department, University Hospital Meyer, Florence, Italy; ^5^ Department of Engineering and Architecture, University of Trieste, Trieste, Italy

**Keywords:** cardiac imaging, strain, MRI, cardiac function, deformation imaging, feature tracking (CMR-FT)

## Abstract

**Background:**

Advancements in cardiac imaging have revolutionized our understanding of ventricular contraction. While ejection fraction (EF) is still the gold standard parameter to assess left ventricle (LV) function, strain imaging (SI) has provided valuable insights into ventricular mechanics. The lack of an integrative method including SI parameters in a single, validated formula may limit its use. Our aim was to compare different methods for evaluating global circumferential strain (GCS) and their relationship with global longitudinal strain (GLS) and EF in CMR and how the different evaluations fit in the theoretical relationship between EF and global strain.

**Methods:**

Retrospective monocenter study. Inclusion of every patient who underwent a CMR during a 15 months period with various clinical indication (congenital heart defect, myocarditis, cardiomyopathy). A minimum of three LV long-axis planes and a stack of short-axis slices covering the LV using classical steady-state free precession cine sequences. A single assessment of GLS on long axis (LAX) slices and a double assessment of GCS and EF with both short axis (SAX) and LAX slices were made by a single experienced CMR investigator.

**Results:**

GCS-SAX and GCS-LAX were correlated (*r* = 0.77, *P* < 0.001) without being interchangeable with a high reproducibility for GCS, GLS and EF. EF calculated from LAX images showed an overestimation compared to EF derived from SAX images of 7%. The correlation between calculated EF and theoretical EF derived from SI was high (*r* = 0.88 with EF-SAX, 0.95 with EF-LAX).

**Data conclusion:**

This study highlights the need to integrate strain imaging techniques into clinical by incorporating strain parameters into EF calculations, because it gives a deeper understanding of cardiac mechanics.

## 1 Introduction

During the past decade, technological progresses in non-invasive cardiac imaging (such as echocardiography and cardiac magnetic resonance) allowed significant improvements in our understanding of the physiology of ventricular mechanical contraction. Thus, nowadays, the ejection fraction (EF), based on a volumetric approach to systolic function analysis, is no longer considered the unique way of expressing the state of left ventricle (LV) function. Nevertheless, in daily practice, EF remains the most used parameter because of its simplicity and its reliability as a prognostic factor (1, 2). The development of deformation imaging, or strain imaging (SI), has led to a better understanding of ventricular mechanics and helped in the understanding of heart failure, especially in cases of diastolic dysfunction with preserved EF (3–5). The SI technology is based on tracking the tissue during the cardiac cycle in transthoracic echocardiography (TTE) by optical flow techniques (Speckle Tracking) (6, 7). The SI was secondary developed in cardiac magnetic resonance (CMR). Initially, various techniques were proposed, including myocardial tagging and strain-encoding (SENC), before the recent advancement of Feature Tracking (FT) technology (8–10). The primary advantage of using SI with CMR is the precision in positioning the short-axis (SAX), allowing for a more accurate assessment of GCS and GRS. This method is less reliant on the operator compared to TTE, thus enhancing its reliability (8, 11). Nowadays, FT has become the most widely used CMR technique as it allows for strain evaluation without the need for additional sequences. It allows the assessment of several parameters including the global longitudinal strain (GLS) describing the base-apex shortening, the global circumferential strain (GCS) describing the reduction of the mean diameter of the left ventricle and the global radial strain (GRS) describing the thickening of the left ventricular wall. In practice, GCS and GRS are less utilized than GLS because more difficult to assess in TTE, mainly due to the challenge of obtaining reproducible short axis slices (SAX). SI may be still underexploited in clinical practice although it is proven early prognostic factors in many cardiomyopathies both in adults and in pediatric populations (12, 13). One explanation could be that until recently, these parameters were evaluated independently of each other, giving therefore only a partial view of a complex mechanism. Indeed, these parameters may reflect the contribution to contraction of different myocardial layers while they both participate in overall cardiac mechanic efficiency (14). Associating deformation parameters and EF in a single mathematical relationship could be a lead to follow as Stokke et al. (15) and Pedrizzetti et al. (3, 16), introduced a concept of objective interdependency between volumetric and mechanical parameters (Central illustration). This new area of non-invasive exploration of cardiac function has the potential to emerge and to give a relevant space for SI in clinical practice (14). However, these theoretical works combine parameters that can be obtained from different modalities (echocardiography, CMR) and from either long-axis slices (LAX) or short-axis slices (SAX); Recommendations in CMR is evaluating LVEF and GCS from classical SAX slices while evaluation of GLS, that requires LAX views, is less frequent. Recently, CMR studies introduced the possibility to evaluate LVEF and the GCS using LAX, as well, by reconstructing the three-dimensional (3D) LV geometry by a combination of multiple LAX recordings (16).

The systematic evaluation and comparison of different FT-CMR measurement methods for these parameters (GCS, GLS, GRS and LVEF) in clinical practice has not yet been performed. This work aims to fill this gap by testing these different procedures during the normal clinical activity in CMR with a heterogeneous population of patient followed for various cardiovascular disease. Specifically, to compare the methods for the evaluation of the GCS and LVEF obtained from multi-slice SAX and tri-plane LAX, and how the different evaluations fit in the theoretical relationship between EF and global strain.

## 2 Material and methods

### 2.1 Studied population

All consecutive patients who underwent a complete CMR from November 2021 to March 2023 in a tertiary CMR reference center were included.

Patients with a univentricular heart and cine images of inadequate quality for evaluation were excluded [representing 25 patients (16%)].

Among the cohort, patients with normal cardiac anatomy and with a normal magnetic resonance imaging (MRI) scan were classified as “normal population”.

### 2.2 CMR study

Every CMR exam was acquired on the same MRI (Philips 1, 5 T, ACHIEVA DSTREAM). Images were acquired with a 32-channel phased-array cardiac coil and a vector electrocardiogram for R wave triggering with a basic standardized acquisition protocol and additional sequences specific to each pathology. A minimum of three LV LAX (two-, three- and four-chamber) planes and a stack of SAX slices covering the entire LV were necessary for our study. All Cine images were realized prior to the administration of contrast (17). The sequence used was a classic Steady state free precession (SSFP) sequence with retrospective gating with the following parameters: 30 phases, slice thickness 6–8 mm, no gap, views per segment according to heart rate, number of excitation 1–4, 45° flip angle, repetition time/echo time equal to 3.5/1.5. It was assumed that the influence of slice thickness was not significant until confined in this range; the orientation was considered more relevant for this study and care was taken to position LAX slices to limit the foreshortening.

All cine images were analyzed by a single CMR investigator using Medis Suite, version 3.0 (Medis BV, Leiden, The Netherlands). GCS, GLS were evaluated using dedicated software (QStrain 1.3.0.79, Medis BV, Leiden, The Netherlands). A blinded second reading was performed by the same investigator for the entire cohort 1 month after the first evaluation. The inter-reader variability was performed using a second blinded reading performed by an experienced investigator for 60% of the cohort selected randomly.

### 2.3 CMR measurements

The reference value of LVEF was calculated using the set of SAX cine images as recommended by the guidelines (EF-SAX) (17, 18). Endocardial contours were performed manually. Papillary muscles and the left ventricular outflow track were included in the ventricular volumes. In addition, the value of EF as obtained by the 3D LV model reconstructed by the three LAX slices (EF-LAX) was calculated for comparison. This EF-LAX is to be considered semi-automatized as the observers systematically performed a control of the position of the mitral valve and apical positions.

All strain measurements were realized using a semi-automatic FT technology. The investigator controlled the position of the mitral valve and the apex on LAX slices and corrected eventually major errors of endocardial and epicardial contouring.

GLS was obtained using a triplane analysis on the LAX images and was performed at the LV sub-endocardial level (GLS^endo^) and on average over the thickness of the myocardium (GLS^myo^) with a global result as the average of the values obtained from each of the three slices.

For the GCS, a double assessment was performed:

(1) Using three SAX slices (basal, mid-ventricular, and apical) and taking their average value of them, called GCS-SAX;
(2) Using the three LAX planes (two-, three- and four-chamber), called the GCS-LAX, to reconstruct the 3D LV shape. GCS is then computed by the average circumferential contraction from base to apex over the 3D LV model.

For the GCS-LAX and GCS-SAX, endocardial and myocardial contours were performed (Supplementary Figure S1) to assess endocardial (GCS-LAX^endo^ and GCS-SAX^endo^) and myocardial (GCS-LAX^myo^ and GCS-SAX^myo^) values.

All values of GCS and GLS values are expressed in negative percentage with a higher negative value describing a better shortening of a given myocardial segment related to its original length. GRS values are expressed in positive percentage.

Lastly, an additional theoretical EF (EF^th^) derived from strain values was also assessed for further comparison (15). This metric considers both GLS and GCS, and was computed according to the following mathematical relationship:

$$EF^{\left(\mathrm{th}\right)}=100-100\times \left(\frac{\mathrm{GL}S^{\mathrm{endo}}}{100}+1\right)\times {\left(\frac{\mathrm{GC}S^{\mathrm{endo}}}{100}+1\right)}^{2}$$

### 2.4 Statistical analysis

Continuous data were described as mean ± SD; the paired Student's *t*-test was used to compare normally distributed continuous variables. Values of *p* below 0.05 were taken to indicate statistical significance. A regression model based on least square minimization was used to identify the existence of linear, proportional, and identity relationships between variables pairs. The goodness of fit and accuracy of relationships were evaluated by the correlation coefficient *r*, covariance of residuals, and Bland-Altman test. The inter and intra-reader variability was evaluated using intraclass correlation coefficients (ICC) of type. ICC values under 0.5, between 0.5 and 0.75, between 0.75 and 0.9, and above 0.9 were indicative of poor, moderate, good and excellent reliability respectively (19). The statistical analyses were performed using MatLab (Mathworks, Natick, MA, USA) ver. 9.7 (2019b) with Signal Processing Toolbox ver. 8.3 and Statistics Toolbox ver. 11.6.

### 2.5 Ethics

The study protocols were approved by the local Ethics Committees, IRB 534/21, and complied with the European general data protection regulation (GDPR).

## 3 Results

### 3.1 Population

159 exams were performed during the period. 134 patients reached the inclusion criteria leading to the analysis of 134 CMR exams (84% of the population). Clinical indications for CMR were classified into four groups: acute myocarditis (27/134, 20%), ventricular arrhythmia (21/134, 15.5%), cardiomyopathy (30/134, 22%) and congenital heart disease (56/134, 42%). Among these 134 exams, 34 (25%) were concluded normal with the main indication of suspicion of myocarditis. All patients were in sinus rhythm. The population characteristics and functional parameters are described in Table 1.

**Table 1:** Patients characteristics and CMR measurements.

Parameter	Patients (*n* = 134)	
General		
Age, years (±SD)		18.6 (±11)
Ratio Male/Female		85/49
Weight, Kg (±SD)		56 (±18)
Height, cm (±SD)		1.61(±0.18)
BSA, kg/m^2^ (±SD)		21.3 (±0.35)
CMR indication, *n* (%)	Myocarditis	27 (20)
	Cardiomyopathy	30 (22)
	Congenital heart disease	56 (42)
	Arrythmia	21 (15.5)
CMR results, *n* (%)	Normal	35 (26)
	Myocarditis, pericarditis	12 (9)
	Right side CHD	20 (15)
	Left side CHD	21 (15.5)
	Shunt (VSD, ASD)	9 (6.7)
	Duchenne, Becker cardiomyopathy	8 (6)
	Hypertrophic cardiomyopathy	7 (5.2)
	Dilated cardiomyopathy	8 (6)
	Other	14 (10.4)
Left ventricle LGE, *n* (%)		17 (12.5)
Short axis CMR parameters		
LVEF-SAX, % (±SD)		58 (±7)
RVEF-SAX, % (±SD)		58 (±9)
Stroke volume, ml	Left ventricle	83
	Right ventricle	76
Correlation coefficient between Right and Left SV in normal population		0.94
Indexed LVEDV, ml/m^2^ (±SD)		87 (±30)
Indexed LVESV, ml/m^2^ (±SD)		36 (±21)
Indexed LV mass, gr/m^2^ (±SD)		55 (±18)
GCS-SAX^endo^, % (±SD)		−30.5 (±5.6)
GRS-SAX, % (±SD)		86 (±27)
Long axis CMR parameters		
LVEF LAX, %		65 (±8)
GLS-LAX^endo^, % (±SD)		−27.4 (±5.8)
GCS-LAX^endo^, % (±SD)		−33 (±5.6)
GRS-LAX, % (±SD)		79 (±22)

BSA, body surface area; CMR, cardiac magnetic resonance; CHD, congenital heart disease, VSD, ventricular septal defect; ASD, atrial septal defect; LVEF, left ventricle ejection fraction, RVEF, right ventricle ejection fraction; SAX, short axis; LAX, long axis; SV, stroke volume; GCS, global circumferential strain; GRS, global radial; LVEDV, left ventricle end diastolic volume; LVESV, left ventricle end systolic volume; GLS, global longitudinal strain.

Data are expressed in number or percentage with, when relevant, standard deviation (SD).

All functional parameters examined in the study, including LVEF using a semi-automated LAX method, were successfully obtained in 100% of the patients, even in those with altered LV morphology such as in congenital heart disease and cardiomyopathy.

### 3.2 Inter and intra-observer variability

Concerning the LAX parameters, the intra and inter-observer variabilities were good:

The intra and inter-observer reproducibility of the GLS was excellent with an ICC of 0.9 and 0.86 (*p* < 0.05) for MyoGLS and 0.85 and 0.83 (*p* < 0.05) for EndoGLS respectively.

The LVEF was also very reproductible with an ICC of 0.9 and 0.95 (*p* < 0.05) respectively.

The intra and inter reproducibility of the GCS was good when calculating with LAX slices especially with endocardial contouring with an ICC of 0.83 and 0.94 (*p* < 0.05) for EndoGCS respectively.

The intra observer reproducibility of the GCS was good when calculating with SAX slices especially with myocardial contouring with an ICC of 0.9 (*p* = 0.8) for MyoGCS and an ICC of 0.7 (*p* = 0.07) for EndoGCS.

Concerning the Global Radial Strain, the inter and intra-variability agreement were poor (ICC < 0.5) and therefore is not included in the data exposed in this study.

### 3.3 Relationship between EF-SAX and EF-LAX

**Figure 1:**
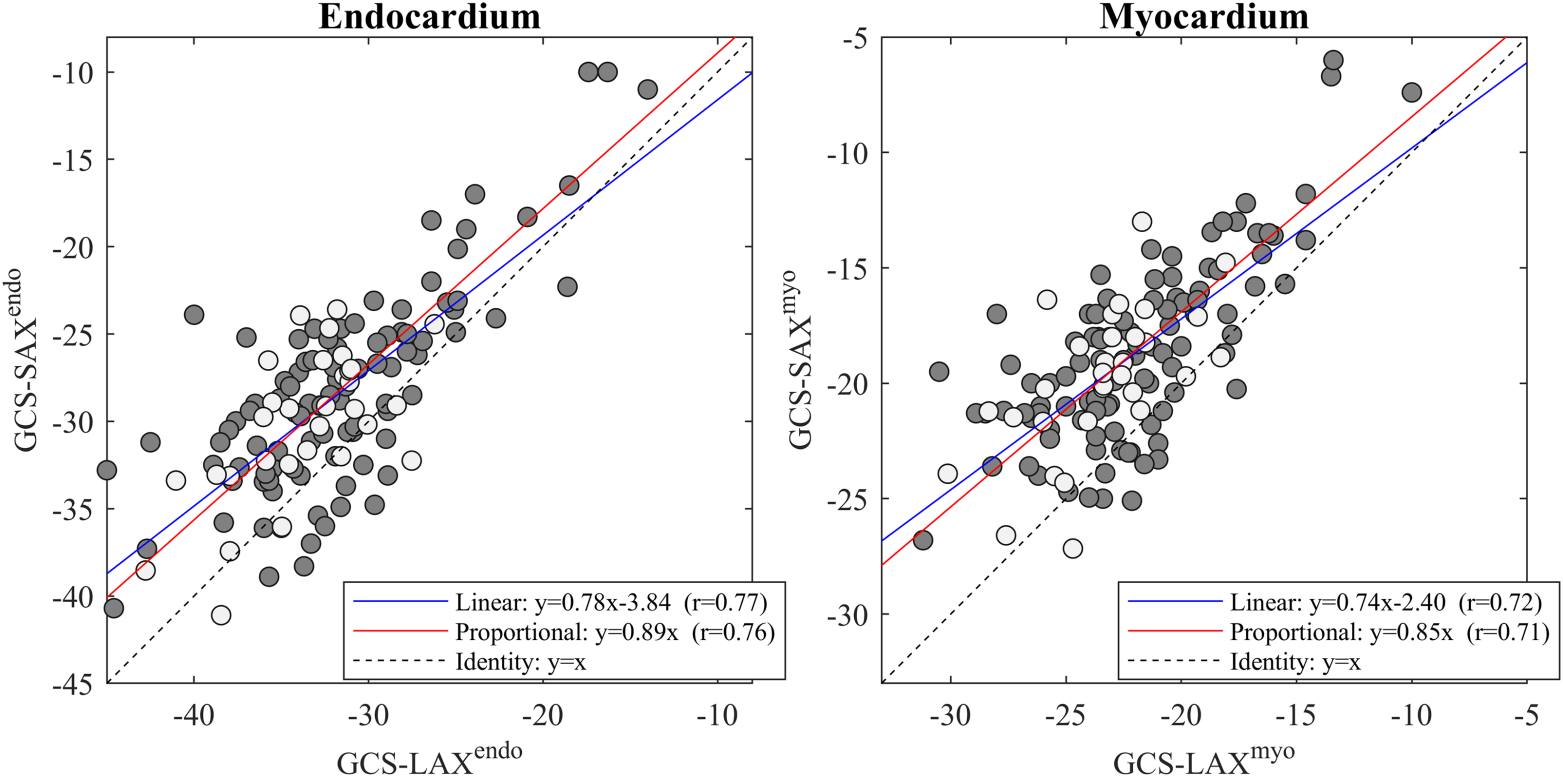
Relationship between EF-LAX and EF-SAX. Direct comparison (left panel) that reports the linear relationship in blue and the proportional relationship in red, and the corresponding Bland-Altman plot (right panel); light-gray points represent the normal group. Correlation is good, however, EF-Triplane presents a systematic overestimation of about 7% with respect to EF-SAX. EF, ejection fraction; LAX, long axis slices; SAX, short axis slices.

**Figure 2:**
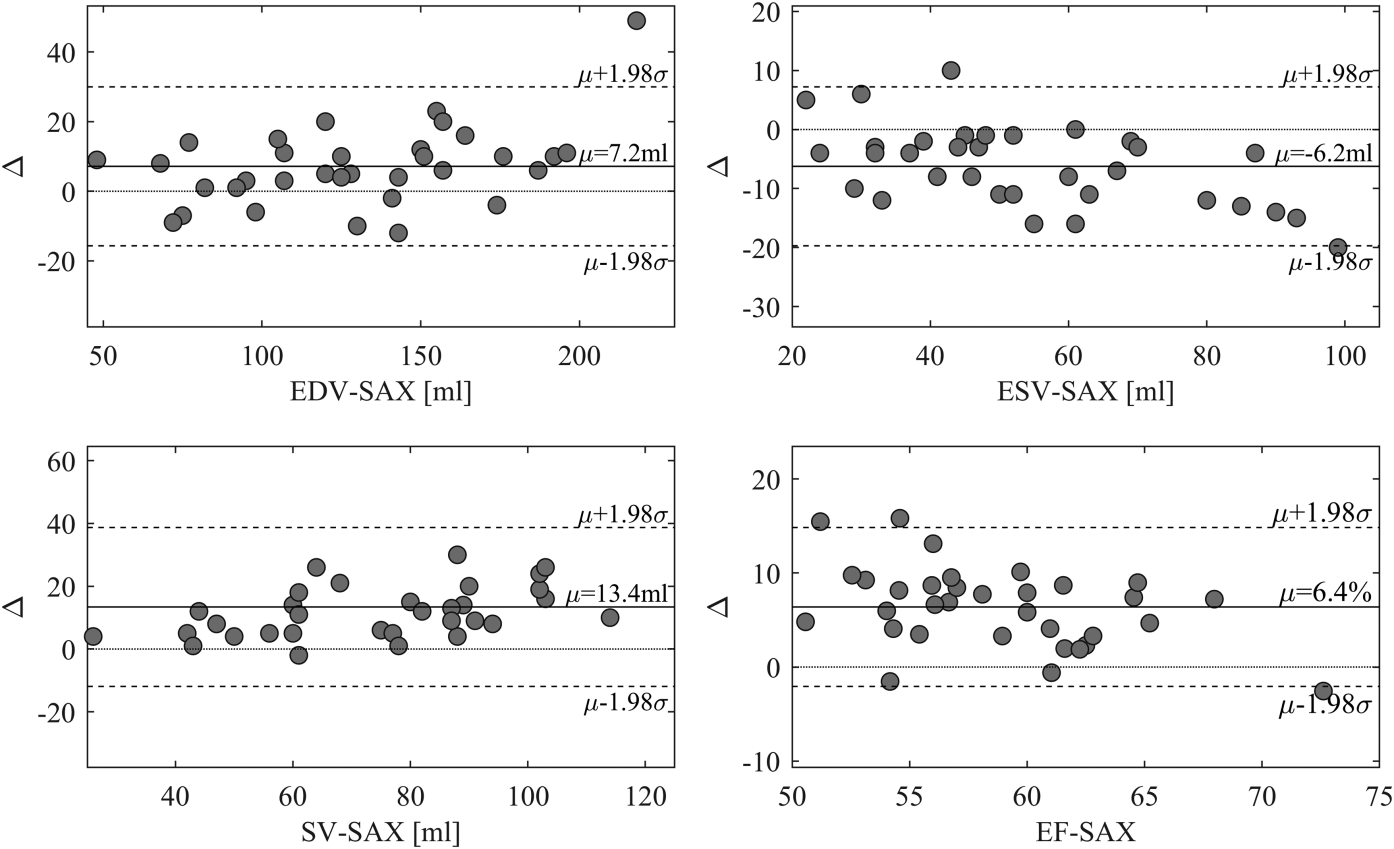
Difference between volumetric measures computed from triplane LAX slices and SAX slices. Bland-Altman plot of the differences between volumetric measures computed from triplane LAX slices and SAX slices in the normal group. LAX calculation presents an overestimation of EDV and an underestimation of ESV, resulting in larger stroke volume (SV) and EF. EDV, end-diastolic volume; ESV, end-systolic volume; LAX, long axis slices; SAX, short axis slices.

EF-LAX presented a good correlation with EF-SAX as shown in Figure 1; nevertheless, the EF-LAX presented a systematic overestimation of about 7% in absolute value with respect to EF-SAX. The global overestimation of the EF-LAX was imputable to a systematic overestimation of the LV end-diastolic volume (EDV) and an underestimation of the LV end-systolic volume (ESV); their combination resulted in a higher stroke volume (SV) and EF. This is shown in Figure 2 for the group of normal population.

### 3.4 Relationship between GCS computed from SAX and LAX projections

**Figure 3:**
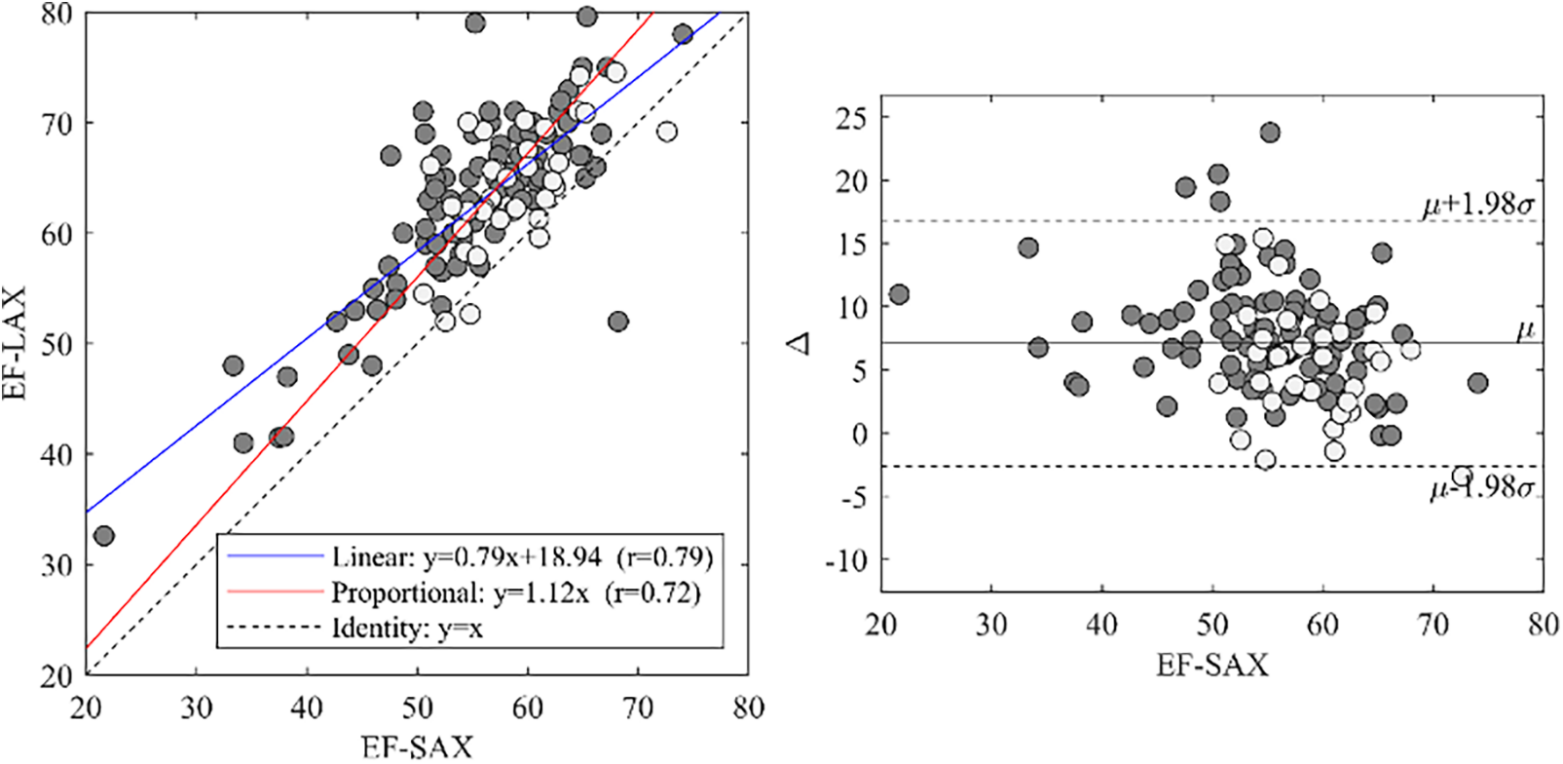
GCS-SAX and GCS-LAX relationship. Relationship between GCS measured from the 3D geometry reconstructed from three LAX slices and measured from SAX slices both at the endocardium (left) and myocardium (right). The general linear relationship is shown in blue and the proportional relationship in red; light-gray points represent the normal group. The correlation between GCS-LAX and GCS-SAX is good, however, the latter presents an underestimation with respect to the former. GCS, global circumferential strain; GLS, global longitudinal strain; LAX, long axis slices; SAX, short axis slices.

The two values of GCS were closely related (*p* < 0.001 with a high correlation coefficient). However, their values were not interchangeable. The linear relationships (Figure 3) shows that the GCS from SAX was systematically lower (about 10% difference relative to the LAX GCS^endo^ values, and 15% for LAX GCS^myo^ values).

### 3.5 Correlation between EF (SAX and LAX) and GLS or GCS (SAX and LAX)

**Figure 4:**
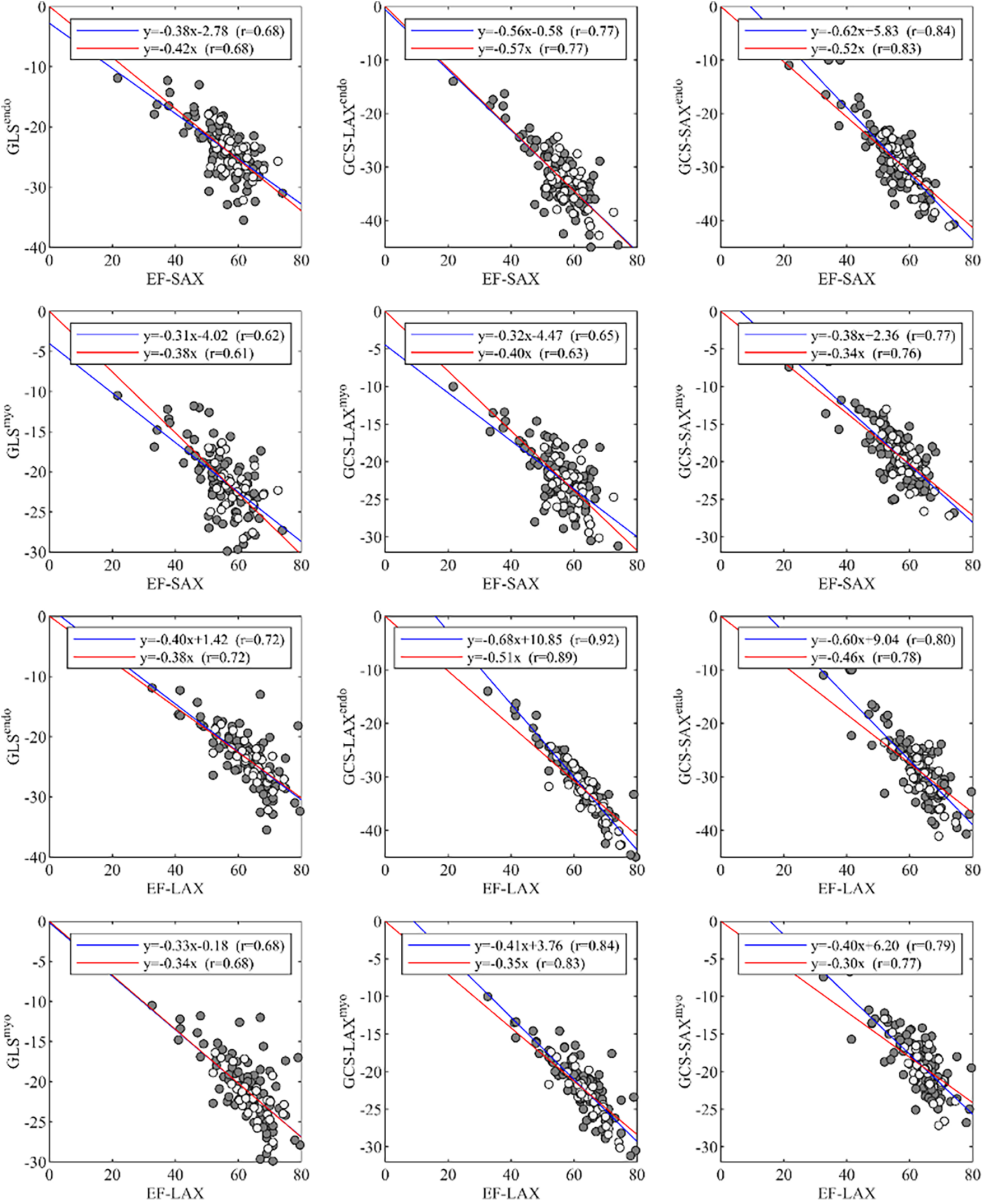
Correlations of global strain values, GLS and GCS, with EF, measured from either LAX or SAX. EF, ejection fraction; GCS, global circumferential strain; GLS, global longitudinal strain; LAX, long axis slices; SAX, short axis slices.

A summary of correlations between EF and global strain is reported in Figure 4. The correlations between EF-SAX and GLS^endo^ (*r* = 0.68) were good, improved with GCS-LAX^endo^ (*r* = 0.77), and highest with GCS-SAX^endo^ (*r* = 0.84). The same correlations were lower when strain was computed on average over the myocardium; When the same correlations were evaluated using EF-LAX, results improved for both GLS^endo^ (*r* = 0.72) and GCS-LAX^endo^ (*r* = 0.92) while it remained similar for the GCS-SAX^endo^ (*r* = 0.80).

### 3.6 Reliability of the theoretical EF^(th)^ in relation to EF (SAX or LAX)

**Figure 5:**
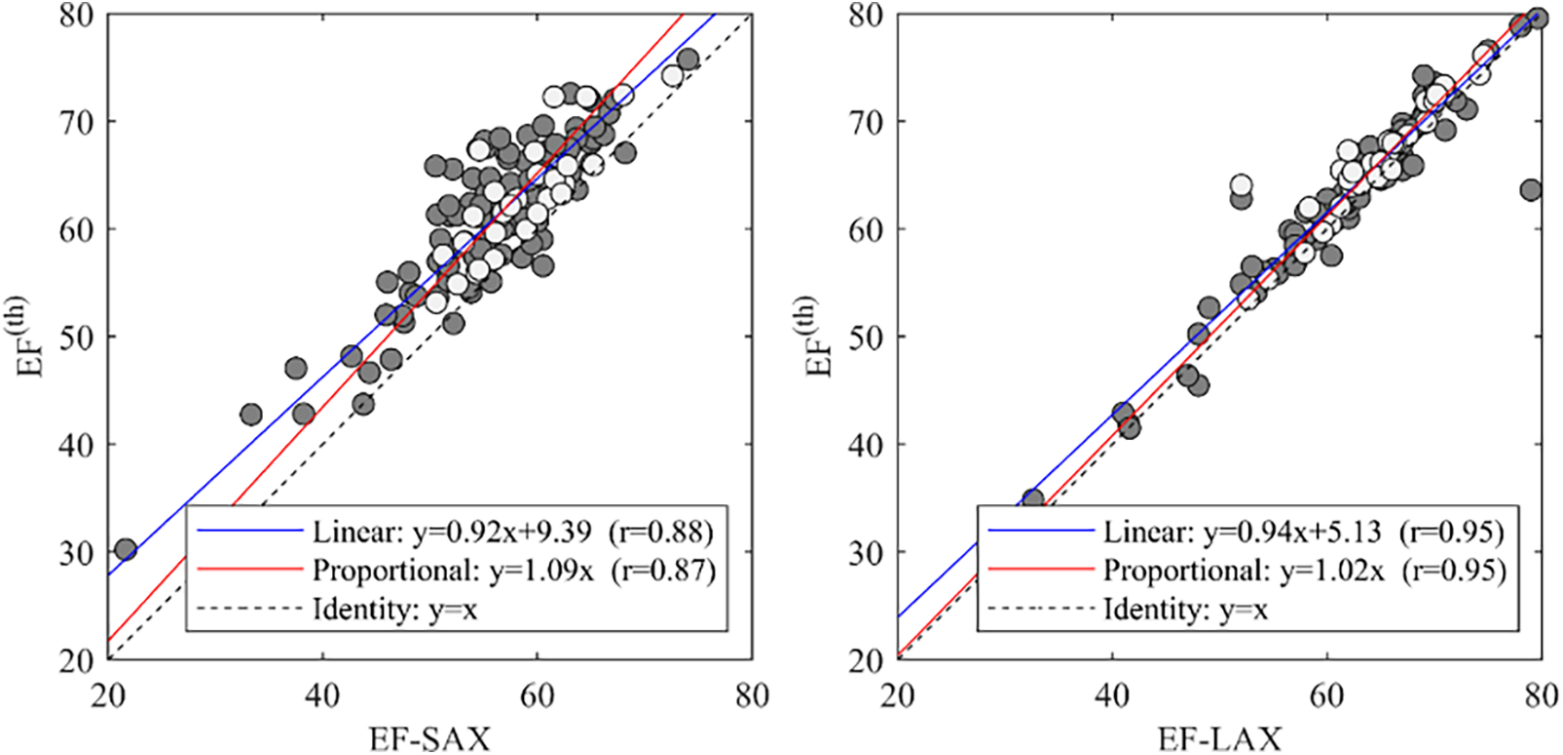
Correlations between EF with the theoretical value EF(th). and EF-SAX. EF, ejection fraction; EF(th), theoretical ejection fraction; SAX, short axis slices.

Using the theoretical relationship (EF^(th)^) described in the method section we compared the EF-SAX with EF^(th)^ computed by inserting GLS^endo^ and GCS-SAX^endo^ in the formula, and EF-LAX with the same using GLS^endo^ and GCS-LAX^endo^. This relationship was well verified in the present dataset (Figure 5); the EF^(th)^ presented a good correlation with EF-SAX (*r* = 0.88), although the theoretical value slightly overestimated EF of about 5% in absolute value. The correlations increased (*r* = 0.95) with EF-LAX with no systematic bias, when both global strain values were obtained from the same set of LAX images.

## 4 Discussion

In the present study, which spanned one year and involved 134 patients, we illustrate the feasibility and reproducibility of CMR-derived SI parameters across a diverse spectrum of left ventricular (LV) morphologies in a heterogenous population.

Based on a systematic comparison between CMR measurements of LV function obtained by SAX and LAX slices performed with FT, we showed that these SI parameters were highly correlated but were not quantitatively interchangeable. Our work, performed in the setting of a heterogeneous population, intends to guide clinical practice by comparing the different techniques of acquisition of GCS, GLS, GRS and EF so as to ensure a strongest accuracy when employing different methods. This approach seems us important as functional parameters are used in daily practice as prognostic outcomes and can have an impact on therapeutic decision (20).

First of all, concerning EF calculation, the gold standard in CMR is still the manual SAX method (21). Semi-automatized EF-LAX performed with FT technology seems to systematically underestimate the ESV and overestimate the EDV and in consequence the EF by 7% in relative terms. This is in line with previous reports evaluating volumes and EF with CMR or TTE using manual or semi-automatized LAX method (21–25).

It is obvious that employing a semi-automated LAX method could result in substantial time savings. However, like any automated software, it does not fully consider the intricacies of abnormal left ventricle geometries. Indeed, in our cohort we faced two “risky” situations concerning almost the third of our cohort: in cardiomyopathy, the systolic borders can be harder to delimit in LAX than in SAX plane especially in systole where the papillary muscles can merge with the myocardial walls. Moreover, in congenital heart disease the left ventricle outflow track often has a complex geometry that has to be included in the 3D volume, which is not easily feasible with automated FT measures (26). Hence, we assumed that expert-performed manual contouring was the most reliable method for assessing volumes in especially in hypertrophied or twisted ventricles (27). However, this level of difference could be also explained by an insufficient manual correction of the left ventricle boundaries but the excellent correlation of EF-LAX in inter and intra-observer measures seems to exclude this theory. Finally, it depends also on the quality of the acquisition planes itself, and the precision of their 3D position relative to the ventricular anatomy.

**Figure 6:**
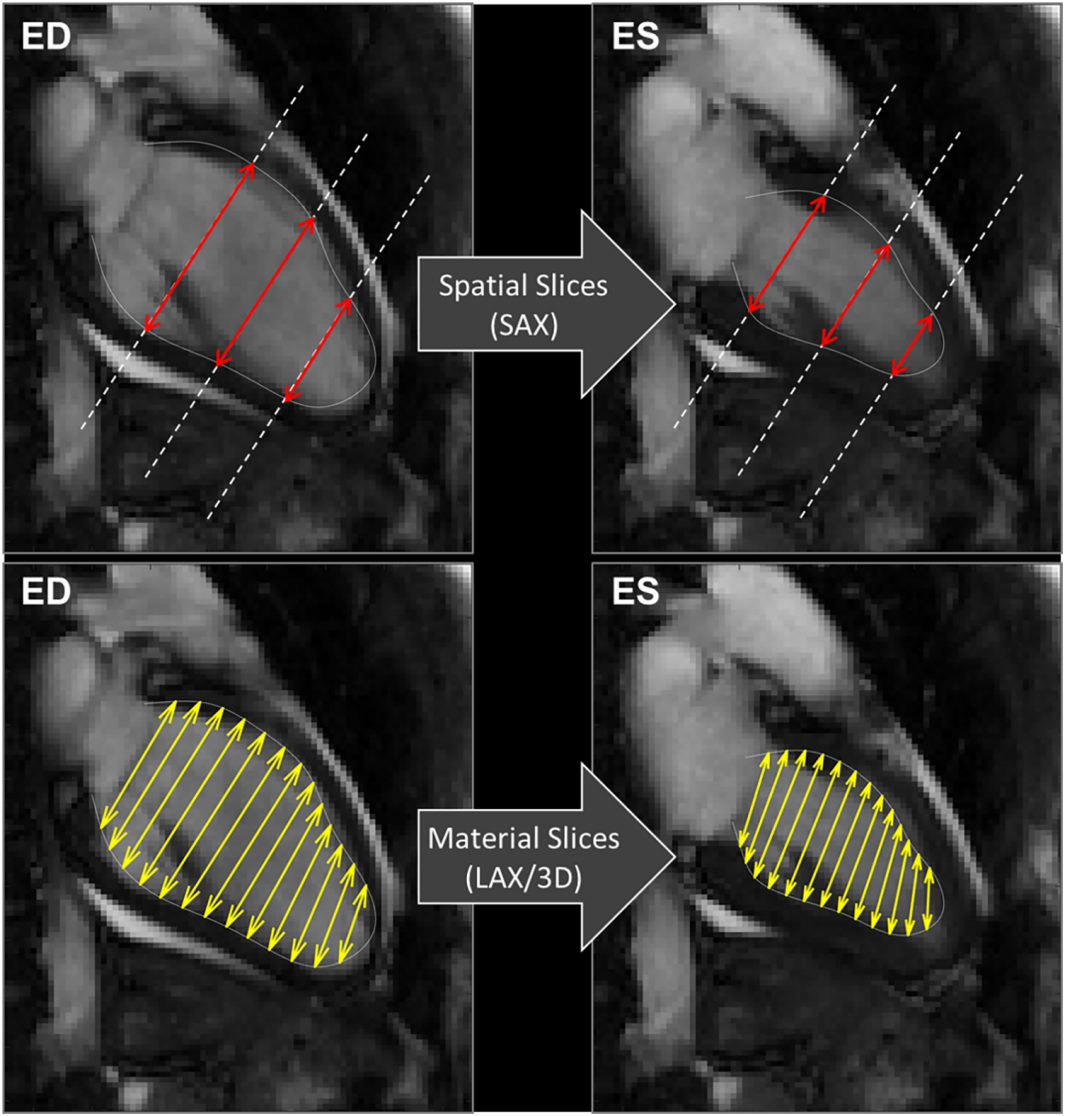
CMR 2 chamber views. Exemplifying the computations of GCS, explaining the differences between the usage of spatially fixed SAX slices (above) and material slices in a moving 3D model (below). GCS, global circumferential strain, SAX, short axis slices.

Concerning the method of acquisition of GCS, we confirmed that GCS-SAX is systematically lower than GCS-LAX (16). This difference follows from the fact that GCS in SAX is taken on transversal slices that remain fixed in space while the LV contracts. As exemplified in Figure 6, during contraction the base moves apically and slices that are located at the LV base at end-systole became more apical at end-diastole. Therefore, a more basal and wider portion of myocardium enters the fixed slice, thus introducing an apparent widening and reducing the GCS. Differently, when measuring GCS in LAX images, the transversal slices move together with the tissue, thus they can cover the entire LV from base to apex uniformly during the entire heartbeat and give a closer representation of the physical contraction of the LV chamber. This phenomenon was previously mentioned in a very similar proportion between the two GCS measured on the endocardium in a different dataset (16). Of course, these phenomena would theoretically be better tackled in 3D cine sequences, but accessibility and acquisition time are still important limitations for the widespread application of strain in these sequences (24, 28). Finally, the usage rules of GCS with CMR should be clarified as a recent publication of the American society of echocardiography did it for TTE (29). Nevertheless, the fact that normal GCS values are potentially depending on sex, age and race is a barrier to its general utilization in clinical practice (29). Notably, the GRS exhibited poor inter- and intra-observer correlation and lacked association with EF. This discrepancy is likely attributed to challenges in automatically defining the epicardial contour across all SAX slices.

Our findings reveal strong correlations between individual global strain parameters and EF. This is in agreement with previous studies (30–33), confirming that GCS and GLS are metrics of the contractile function. This is particularly true when considering endocardial borders, as already described with several SI techniques (34–38). Indeed, EF calculation is based on endocardial displacements, which are more directly related to endocardial deformations. However, LVEF is imputable to both longitudinal shortening and transversal thickening; therefore, the correlation of LVEF with a single strain component is necessarily, incomplete, whereas each component can reflect different aspects of systolic function or dysfunction (9). Accordingly, the higher correlation between EF^(th)^ (including GCS and GLS) and the SAX-EF (result of a volumetric approach of EF) is a strong argument to systematically integrate both for a better understanding of systolic function (3, 16). Although the different layers across the myocardial thickness work in concert and cannot be separated, GLS expresses more the contraction of subendocardial fibers, the first to be impacted in many pathological processes, while GCS corresponds more to medial and subepicardial fibers that are generally impacted at a later stage of the disease (14, 16, 39). Identifying these myocardial transmural patterns can be a way to a better understanding of pathological or adaptative myocardial remodeling.

Nowadays, cardiologists and radiologists are always in search for integrated tools that could give more hemodynamic and functional information in a shorter time. Artificial intelligence enables constant progress especially in cardiology (40). The integration of the EF^(th)^ (including GCS and GLS) in CMR software's could be a way to give access to mechanical and volumetric information in a single click without the need for specific sequence.

Finally, FT-strain imaging provides only limited insights into segmental contraction of both ventricles. Indeed, this sequence appears to be less reliable due to significant variations that depend on the spatial resolution which depend on the cardiac frequency and the number of phases of the cine sequences (41). Among the various available techniques, the Strain-encoded sequence, has demonstrated superiority over FT in assessing segmental myocardial wall motion and has comparable performance in terms of reproducibility (42). Technical advance has made possible its acquisition within a breath hold (fast-SENC) making this sequence equally usable in clinical practice than FT (43). Furthermore, it has been established as a reliable prognostic indicator in heart failure, coronary artery disease, transplant vasculopathy (44–46) and enhance the performance of CMR stress testing (47). On the other hand, inward displacement emerges as a promising and complementary approach to FT strain, given its application in cine sequences. However, like any parameter, its efficacy needs to be thoroughly studied across different pathological conditions before being implemented in clinical practice (48, 49).

### 4.1 Study limitations

We recognize both strengths and weaknesses of our study. This is the first study to present a systematic comparison between CMR measurements of LV function including SI with two FT methods in a real-life population. However, the recruitment was made in a congenital heart disease expert center thus our population was not representative of the general population. The population defined as normal in this study cannot be generalized, as it was recruited from a pool of patients with suspected heart disease in whom the CMR exam was negative for tissue or structural abnormalities, but in whom sub-clinical disease might be difficult to completely exclude. Moreover, the single-center, retrospective nature of our study limits our ability to make definitive conclusions.

## 5 Conclusion

Acquisition of CMR strain imaging in a heterogeneous population is feasible and reproducible—with an exception for the SAX radial strain—across a wide range of LV morphologies. These measurements have a good correlation with the gold-standard SAX derived LVEF. Finally, the reported reliability of the theoretical relationship between global strain and volumetric changes supports their conceptual integration and paves the way for a more comprehensive assessment of myocardial pathologies but still warrants further studies.

## Data Availability

The raw data supporting the conclusions of this article will be made available by the authors, without undue reservation.

## Supplementary material

Supplementary Figure S1 CMR in short axis view with endocardial and epicardial contours to the calculation of GCS^endo^ and GCS^myo^. A: anterior; I: inferior. Yellow contours: epicardial borders; Blue contours: endocardial borders.

Supplementary Figure S2 Methods of acquisition of ventricular End-Fraction and strain. RV, right ventricle; LV, left ventricle; LA, left atrium; LVEF, left ventricle end-fraction; EDV, end-diastolic volume; ESV, end-systolic volume; GCS, global circumferential strain, GLS, global longitudinal strain.
