# Supplementary figures and images for: Comparative analysis of left ventricle function and deformation imaging in short and long axis plane in cardiac magnetic resonance imaging
Source: Front Cardiovasc Med. 2024 May 2;11:1388171. doi: 10.3389/fcvm.2024.1388171 (PMC11097778; doi:10.3389/fcvm.2024.1388171)

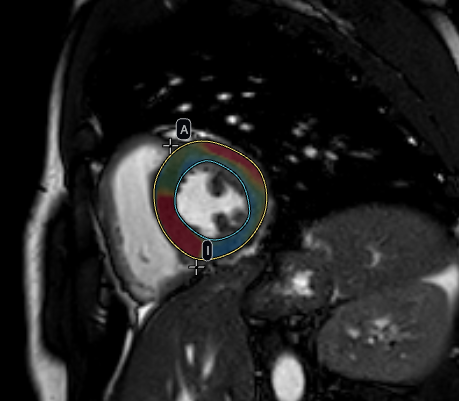

Supplement: Supplementary Figure S1 — CMR in short axis view with endocardial and epicardial contours to the calculation of GCSendo and GCSmyo. A: anterior; I: inferior. Yellow contours: epicardial borders; Blue contours: endocardial borders. [file Image1.tiff]

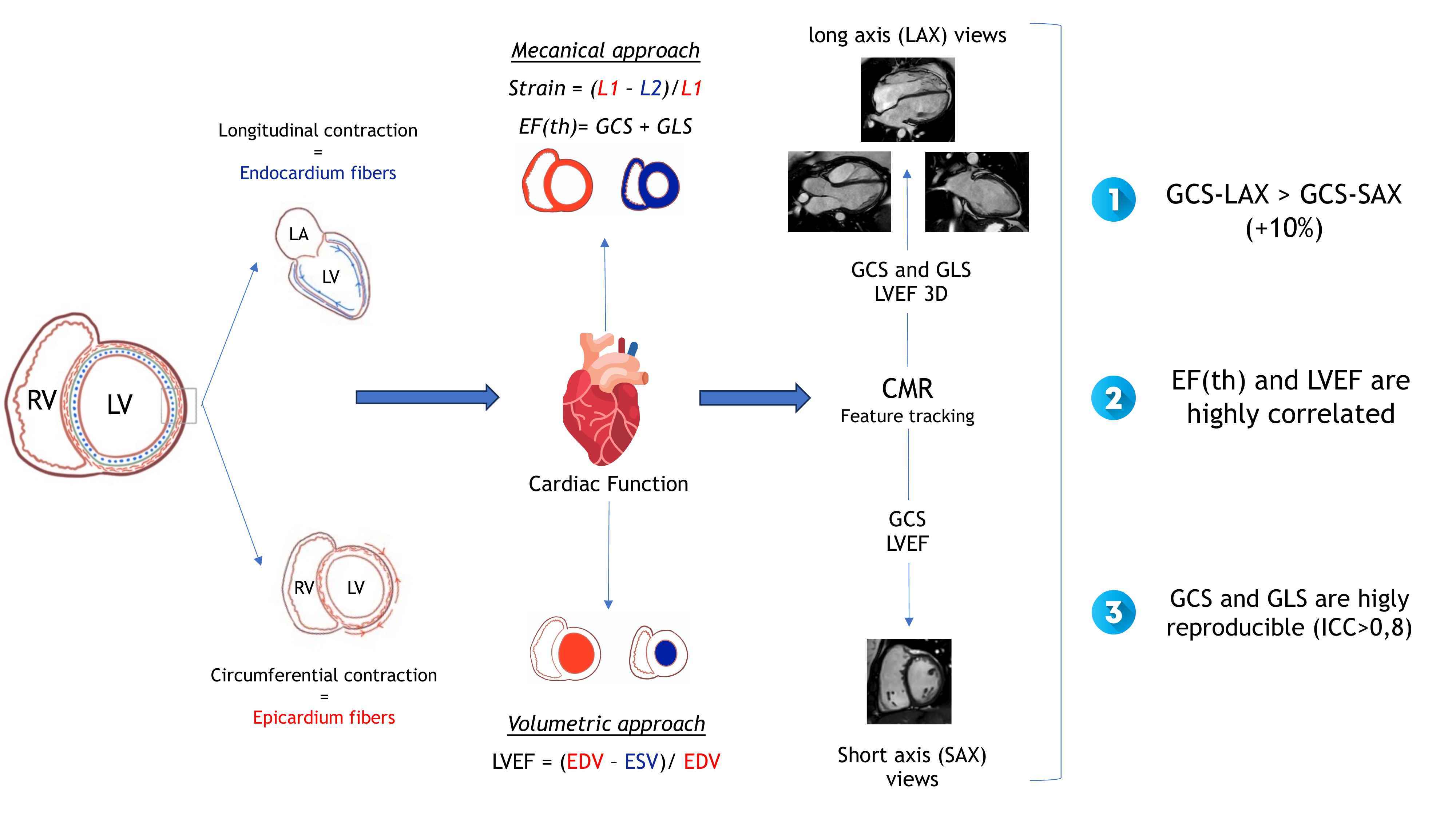

Supplement: Supplementary Figure S2 — Methods of acquisition of ventricular End-Fraction and strain. RV, right ventricle; LV, left ventricle; LA, left atrium; LVEF, left ventricle end-fraction; EDV, end-diastolic volume; ESV, end-systolic volume; GCS, global circumferential strain, GLS, global longitudinal strain. [file Image2.jpeg]
